# IOP Maintenance in SLT-treated Eyes following Subsequent Phacoemulsification and IOL

**DOI:** 10.5005/jp-journals-10008-1131

**Published:** 2013-01-15

**Authors:** Ejaz Ansari

**Affiliations:** Consultant Ophthalmic Surgeon, Maidstone and Tunbridge Wells NHS Trust, Maidstone United Kingdom; Reader, University of Kent at Canterbury, Kent United Kingdom

**Keywords:** Selective laser trabeculoplasty, Cataract Surgery and Glaucoma.

## Abstract

To assess whether the intraocular pressure (IOP) in selective laser trabeculoplasty (SLT)-treated eyes is maintained following subsequent phacoemulsification and lens implant (phaco + IOL).

Retrospective single center review of 45 eyes of 35 patients who had open angle glaucoma (OAG), successfully treated by SLT by the same surgeon (EA), and then had routine phaco + IOL by same surgeon (EA).

The main outcome measures were baseline (SLT-treated) IOP and IOP at 3, 6 and 12 months following subsequent routine phaco + Intraocular lens (IOL). Secondary outcome measures were visual acuity pre- and post (phaco + IOL) and any complications.

The study found that IOP reduction with SLT is not significantly affected by subsequent phaco + IOL in patients with OAG.

**How to cite this article:** Ansari E. IOP Maintenance in SLT-treated Eyes following Subsequent Phacoemulsification and IOL. J Current Glau Prac 2013;7(1):17-18.

## INTRODUCTION

The aim of this study was to assess the intraocular pressure (IOP) control following small incision cataract surgery and lens implant in cases of open angle glaucoma (OAG) with well-controlled IOP previously achieved with selective laser trabeculoplasty (SLT) alone. This would provide valuable information to the surgeon as to the likelihood of further IOP control that might be required after phaco + IOL and it would be helpful in informing the SLT-treated patient with OAG more fully prior to phaco + IOL.

SLT has been established for over a decade as an effective treatment modality for lowering IOP in OAG patients.^1-3^ It is safe and potentially repeatable and can be considered in those patients who cannot tolerate drops or whose IOP is not controlled with drops sufficiently well.^1^

Phaco + IOL is well-established as the main surgical procedure for cataract in centers worldwide. It is associated with IOP changes postoperatively in both OAG^4,5^ and angle closure glaucoma (ACG),^6^ although ACG patients benefit significantly more than OAG patients with respect to sustained postphaco IOP reduction.^5^

Cataract frequently coexists in patients with OAG. It is important to ascertain, whether the IOP would be affected adversely in patients previously treated with SLT, if they have phaco + IOL subsequently.^7,8^ This is particularly pertinent, when informing patients who are obviously curious if their IOP control without drops would suffer following subsequent phaco + IOL. Patients whose IOP is controlled without drops following SLT might be faced with more laser treatment or treatment with drops, if the IOP were to increase significantly following phaco + IOL. We are not aware of any studies addressing the issue of IOP control in SLT-treated patients after phaco + IOL.

## MATERIALS AND METHODS

Retrospective single center review of 45 eyes of 35 patients who had OAG successfully treated by SLT by the same surgeon (EA) and then had routine phaco + IOL by same surgeon (EA). There were 15 male and 20 female patients, with mean age (SD) of 75.5 years (11.2). All patients had had SLT first-line for the treatment of OAG. No patients were taking ocular hypotensive drops in addition. Time between SLT and phaco + IOL ranged between 2 and 36 months. IOP was measured at 3, 6 and 12 months following phaco + IOL.

Statistical analysis was performed using the paired t-test for comparison between baseline and postoperative IOP in operated eyes. All statistical tests were completed at a 5% level of significance.

**Table 1:** **Table 1:** IOP (SD) levels during the course of the study

*IOP in mm Hg at baseline (SD)*		*IOP in mm Hg at 3 months (SD)*		*IOP in mm Hg at 6 months (SD)*		*IOP in mm Hg at 12 months (SD)*	
15.8 (1.8)		13.9 (1.5)		14.3 (1.7)		14.8 (1.8)	

## FINDINGS

**Graph 1:**
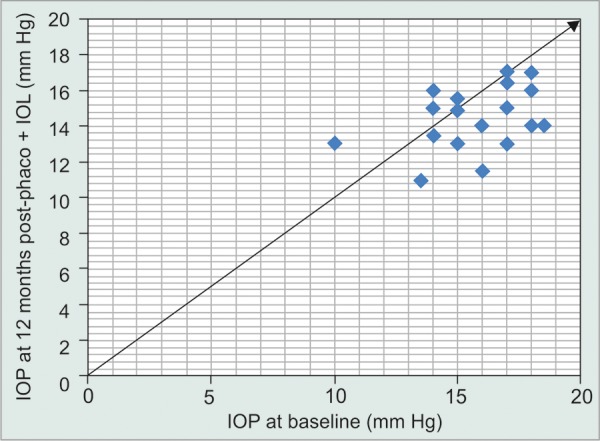
Scatterplot of baseline IOP and IOP 12 months after phaco + IOL

The main outcome measures were baseline (SLT-treated) IOP and IOP at 3, 6 and 12 months following routine phaco + IOL. Secondary outcome measures were visual acuity pre-and post-(phaco + IOL) and any complications. The mean baseline IOP (SD) was 15.8 mm Hg (1.8). At 3, 6 and 12 months post-(phaco + IOL), the mean IOP (SD) were 13.9 mm Hg (1.5), 14.3 mm Hg (1.7), 14.8 mm Hg (1.8; Table 1). All cases were routine with mean preoperative visual acuity of 6/18, improving to 6/9 + 2 unaided at 12 months. Graph 1 is a scatterplot showing the IOP at 12 months following phaco + IOL compared to baseline levels.

## DISCUSSION

All our patients had 360° of SLT as first-line treatment for POAG. All patients had been well controlled following one SLT session with similar IOPs at baseline. At each time point following subsequent phaco + IOL, the IOP was reduced and this was maintained for the 12-month period of follow-up.

The interval period between SLT and phaco + IOL did not affect subsequent IOP control. It was very encouraging that even those cases where SLT had been performed 2 to 3 years earlier, the IOP control was sustained following subsequent phaco + IOL. It is known that inflammatory mediators, including cytokines, are upregulated following SLT.^9^ There is evidence for this also occurring following cataract extraction and intraocular surgery generally, and this might explain the additional sustained IOP reduction that was observed following subsequent phaco + IOL.^10,11^

Our study is limited by the small sample size and the retrospective nature of data collection. A larger study could be conducted prospectively, perhaps comparing the persistence of IOP reduction in SLT-treated patients undergoing phaco + IOL and those who do not have any intraocular surgery. For example, does subsequent phaco + IOL give a boost to IOP reduction that lasts for a period of time beyond what would be expected following SLT alone?

The results of this study should encourage both surgeons and patients that IOP control will be sustained in SLT-treated OAG cases following subsequent phaco + IOL. It is a useful clinical point and a reassuring fact that can be used to inform SLT-treated patients more fully prior to routine phaco + IOL.
